# Letter to the editor regarding “Two-stage laparoscopic transversus abdominis plane block as an equivalent alternative to thoracic epidural anaesthesia in bowel resection—an explorative cohort study”

**DOI:** 10.1007/s00384-024-04641-8

**Published:** 2024-05-02

**Authors:** Murat Izgi, Betul Basaran

**Affiliations:** 1 Hacettepe University, Ankara, Turkey; 2 Karamanoglu Mehmetbey University, Karaman, Turkey

Dear Editor:

We read with great interest the recently published article that compares the effects of two-stage laparoscopic transversus abdominis plane block (TS-L-TAPB) with thoracic epidural anesthesia (TEA) and one-stage L-TAPB (OS-L-TAPB) at the postoperative period in patients undergoing elective laparoscopic bowel surgery [1]. We’d like to offer some critique on this fascinating issue. Optimal postoperative pain management allows for early ambulation and rehabilitation, which improves the quality of recovery following colorectal surgery. Multimodal analgesia, including paracetamol and nonsteroidal anti-inflammatory medications, can be utilized in conjunction with local portal site infiltration or interfascial plane blocks, such as transversus abdominis plane block, to reduce opioid use [2]. Nonetheless, the pharmacological components of multimodal analgesia should be administered before patients experience pain in order to maximize their benefits [3]. The authors did not administer the non-opioid medications for multimodal analgesia at the indicated time. This may lead to a bias among the three study groups. Furthermore, Enhanced Recovery After Surgery (ERAS) procedures use a combination of paracetamol and nonsteroidal anti-inflammatory drugs to alleviate postoperative discomfort. The pain treatment protocol presented in this article is not arranged in accordance with the ERAS recommendations. The pain management approach used may make it difficult to compare the outcomes to the literature. Furthermore, between postoperative days 1 and 3, the authors stated that TS-L-TAPB produces a clinically significant difference when compared to the TEA and OS-L-TAPB groups. As far as we know, no investigation has implicated the minimum clinically significant difference in morphine intake in colorectal surgery. Therefore, it is difficult to make this conclusion. Finally, there was no methodologic description of anesthetic management in patients undergoing laparoscopic colorectal surgery. Because total intraoperative opioid consumption may affect early postoperative pain scores with early postoperative morphine consumption without reporting intraoperative opioid consumption, comparing the three study groups for analgesia would not yield objective results [4].

Based on the foregoing, establishing pain management studies using ERAS-recommended analgesic protocols and reporting anesthetic management protocols may yield more accurate results when using TS-L-TAPB over TEA and OS-L-TAPB.

## Data Availability

No datasets were generated or analyzed during the current study.
